# Supramolecular Chemistry in Metal–Organic Framework Materials

**DOI:** 10.1002/adma.202414509

**Published:** 2025-02-02

**Authors:** Eugenia Miguel‐Casañ, Georgia R. F. Orton, Danielle E. Schier, Neil R. Champness

**Affiliations:** ^1^ School of Chemistry University of Birmingham Edgbaston Birmingham B15 2TT UK

**Keywords:** metal–organic frameworks, reticular chemistry, supramolecular chemistry

## Abstract

Far from being simply rigid, benign architectures, metal–organic frameworks (MOFs) exhibit diverse interactions with their interior environment. From developing crystal sponges to studying reactions in framework materials, the role of both supramolecular chemistry and framework structure is evident. We explore the role of supramolecular chemistry in determining framework…guest interactions and attempts to understand the dynamic behavior in MOFs, including attempts to control pore behavior through the incorporation of mechanically‐interlocked molecules. Appreciating and understanding the role of supramolecular interactions and dynamic behavior in metal–organic frameworks emerge as important directions for the field.

## Graphical abstract

This review highlights synergies between reticular chemistry and supramolecular chemistry. The role of supramolecular interactions in determining framework…guest interactions and attempts to understand dynamic behavior in metal–organic frameworks (MOFs), particularly emphasizing the development of crystal sponges, studying reactions in frameworks and attempts to control pore behavior through the incorporation of mechanically‐interlocked molecules, is explored.

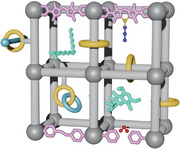

## 1 Introduction

Reticular and supramolecular chemistry strategies provide robust pathways to the design and synthesis of complex, highly‐organized structures.^[^ ^1^ ^]^ Reticular chemistry, the chemistry of linking molecular building blocks by strong bonds to make extended crystalline structures,^[^ ^2^ ^]^ employs the idea of using building‐blocks with specific properties in a modular fashion allows targeted organization of chemical functionality in 3D space, readily demonstrated by the development of metal–organic frameworks (MOFs)^[^ ^3^, ^4^ ^]^ and their use in a wide variety of applications.^[^ ^5^, ^6^, ^7^, ^8^ ^]^ In supramolecular chemistry, widely defined as “chemistry beyond the molecule,”^[^ ^9^ ^]^ control over non‐covalent interactions between molecules permits the formation of larger, organized structures and complex assemblies. Despite clear conceptual links between the ideas of reticular chemistry and those of supramolecular chemistry, there are also clear differences. These differences are most notable in that reticular chemistry specifically employs “strong bonds”^[^ ^1^, ^3^, ^5^, ^6^ ^]^ whereas supramolecular chemistry famously employs intermolecular interactions and weaker, reversible bonds.^[^ ^9^ ^]^

Considering the similarities in the use of a modular building‐block approach, it would be unwise to decouple the concepts of reticular and supramolecular chemistry. Rather there are clear advantages in considering the interplay between these ideas and strategies when designing reticular materials, and MOFs in particular, for specific applications. In this article we discuss the important role that supramolecular chemistry plays in a variety of MOF syntheses and applications, emphasizing the benefits of considering the full palette of chemical interactions, and the full spectrum of strengths of bonds and interactions, when designing and employing reticular materials.

In this review, we explore the role of supramolecular chemistry in the applications of MOFs, with a particular focus on how intermolecular interactions influence host–guest behavior. We begin by briefly examining the influence of supramolecular interactions on guest adsorption, drawing parallels to the use of MOFs as “crystal sponges,” where a greater appreciation of host–guest interactions is crucial. Building on these concepts, we discuss the role of MOFs as scaffolds for supporting chemical reactions, which requires an understanding of the framework pores and their decoration. Finally, we consider how the properties of these framework pores can be considered not only as “empty bottles” but as potentially dynamic environments. In this context, we highlight the integration of mechanically interlocked molecules (MIMs)—a pinnacle of supramolecular chemistry—into MOFs, and how this approach may in the future lead to pores with adaptable host–guest properties. Throughout, we seek to draw parallels between reticular and supramolecular chemistry and emphasize the importance of a holistic perspective on intermolecular interactions in MOF research.

## 2 MOFs as Adsorbents and Crystal Sponges

One of the main focuses of MOF research has employed their porosity to entrap guest molecules. Since the earliest days of the field, it has been apparent that MOFs provide unique environments to host guest molecules. This feature arises due to the ability to manipulate the size, shape, and functionality of the framework pores. These unique and customizable properties have inspired researchers globally to explore the host–guest possibilities provided by MOFs.

**Figure 1:**
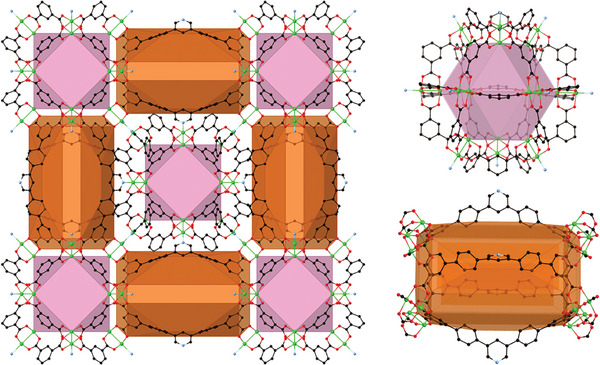
Representations of a MOF (left) in which two different cage‐like pores (shown individually on the right) are observed.^[^ ^10^ ^]^ Molecular cages that form similar pores but within a molecular structure are also reported in the literature.^[^ ^11^, ^12^ ^]^ (Cu, green; O, red; C, black; N, light blue).

The adsorption of guest molecules within MOFs closely mirrors the concepts of host–guest chemistry in supramolecular chemistry, in particular, the ability of cyclic and cage molecules to acts as hosts (**Figure** **1**). As with molecular hosts, the ability of a MOF to host guest molecules in a selective manner relies primarily upon the interactions between host and guest and hence the underpinning concepts of supramolecular chemistry. The synergies between the two fields are evident.

### 2.1 Gas Separation and Adsorption

Supramolecular chemistry plays a pivotal role in enhancing the functionality of MOFs for gas adsorption and separation applications. By leveraging non‐covalent host–guest interactions, the pore environment of a MOF can be tuned to produce materials with superior adsorption capacities and selectivities than traditional adsorbents. Research into the ability of MOFs to trap gases has been inspired primarily by environmental and sustainability targets. Thus, a major focus has been the storage of gases such as H_2_,^[^ ^13^, ^14^ ^]^ CO_2_,^[^ ^13^, ^15^, ^16^ ^]^ and CH_4_,^[^ ^13^, ^17^ ^]^ and for more efficient separation of industrially important gases (e.g., C_2_H_6_/C_2_H_4_/C_2_H_2_).^[^ ^18^, ^19^ ^]^ The importance of MOFs for gas adsorption and separation has been extensively reviewed^[^ ^20^, ^21^, ^22^ ^]^ and we do not seek to revisit the wider field and, therefore, we will focus on some of the key features of guest adsorption in MOFs that rely upon supramolecular chemistry.

**Figure 2:**
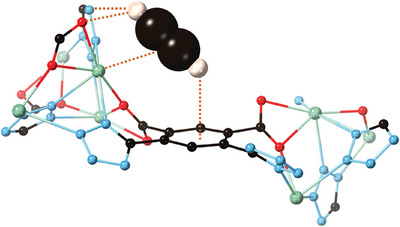
Structural representation of the preferential adsorption site and the various supramolecular interactions observed between C_2_H_2_ and a host MOF as calculated from a GCMC simulation.^[^ ^23^ ^]^ (Ca, light green; O, red; C, black; N, light blue).

An example of how supramolecular approaches can be used to understand and improve gas adsorption is given by a Ca^2+^ based MOF composed of rod‐like SBUs linked by 4,6‐di(1H‐tetrazol‐5‐yl)isophthalic acid ligands.^[^ ^23^ ^]^ The resulting MOF encompasses open hexagonal channels that are rich with open metal sites and organic interaction sites for acetylene (C_2_H_2_) adsorption and recognition. At ambient conditions, the framework showed a high adsorption capacity of 64.6 cm^3^ g^−1^ and strong selectivity for C_2_H_2_ even in binary and ternary mixtures with other hydrocarbons and CO_2_. To better understand this selectivity and the interactions between hydrocarbons and CO_2_ molecules with the framework, Grand canonical Monte Carlo (GCMC) simulations were performed. They found that one end of C_2_H_2_, a C─H group, would form hydrogen bonds with the functional groups of the ligand via C─H…N and C─H…O interactions while the opposing end interacted with the ligand's π‐system. Additionally, the alkynyl unit of ethyne interacted with the Ca^2+^ ion forming M…π bonds (**Figure** **2**). The selective adsorption of ethyne was attributed to the combination of supramolecular interactions observed between guest and framework.

In contrast, the absence of specific host–guest interactions proved to be important for the capture and recognition of CO_2_ from a wet gas stream with the MOF CALF‐20.^[^ ^24^ ^]^ This MOF features fully saturated Zn^2+^ ions bridged by triazolate and oxalate linkers and displayed strong CO_2_ adsorption and selectivity up to 40% relative humidity. Binding‐site modelling using GCMC simulations was used to understand this unusual behavior and identified that the most probable position for CO_2_ guest molecules was in the center of the pores held by attractive dispersion interactions. The same simulations using pure H_2_O, as opposed to CO_2_, as the target guest revealed that uptake of H_2_O was largely guided by the formation of H_2_O clusters. The formation of these clusters starts from an individual H_2_O molecule acting as a seeding point. However, simulations modelling just a single H_2_O molecule in the framework pores showed that weak and non‐specific interactions between H_2_O and CALF‐20 retained the openness of the framework pores allowing for rapid CO_2_ adsorption in industrially relevant environments.

Current CO_2_ capture technology uses aqueous amine solutions despite a variety of drawbacks including high regeneration energies. Incorporation of amine functionalization into MOFs is proving to be a promising alternative. The selective capture of CO_2_ in the majority of these materials is owed to the chemisorption of CO_2_ wherein the molecule is inserted into a MOF‐metal…amine bond to form metal–carbamate chains. The nature of this uptake mechanism limits the capacity to one CO_2_ molecule per pendant amine and some small additional uptake arising from physisorption. Significant increases in CO_2_ uptake capacities can be achieved by designing materials with a mixed adsorption mechanism. A flexible Mg^2+^ based framework, Mg_2_(dobpdc) (dobpdc^4−^ = 4,4′‐dioxidobiphenyl‐3,3′‐dicarboxylate), appended at the nodes with 1‐(2‐aminoethyl)piperidine, exhibits CO_2_ uptake capacity of 1.5 CO_2_ molecules per pendant amine.^[^ ^25^ ^]^ The material exhibits a two stepped adsorption isotherm with the first step accounting for 0.5 CO_2_ molecules per amine being chemisorbed forming ammonium carbamates. The second step is significantly steeper and accounts for an additional CO_2_ molecule per amine. In situ time resolved difference spectra (DRIFTS) revealed that CO_2_ was physisorbed alongside the formation of ammonium carbamate chains indicating synergistic physisorption and chemisorption. A combination of DFT and Rietveld refinements of powder X‐ray diffraction data (PXRD) data placed the physisorbed CO_2_ molecules in the pocket formed from adjacent ammonium carbamates. Furthermore, the position of the CO_2_ molecule revealed that its carbon atom was involved in stabilizing interactions with the oxygen atoms of the neighboring carbamates. These supramolecular interactions drove the physisorption process that resulted in an additional 50% uptake capacity for CO_2_.

An area of increasing interest has been the use of MOFs to trap water, particularly for water harvesting and indoor humidity control.^[^ ^26^, ^27^, ^28^, ^29^, ^30^, ^31^ ^]^ Water entrapment by MOFs provides elegant examples of the use of supramolecular chemistry, particularly framework…H_2_O hydrogen bonding, to improve harvesting performance. A common feature to MOFs with good water adsorption capability is the presence of hydrogen‐bond donor groups on the surface of framework pores. A class of MOFs that have been widely studied are those based on Zr oxoclusters which, in some instances, present hydroxyl or water ligands, both capable of acting as hydrogen bond donors. For example, μ_3_‐OH ligands in the Zr clusters present in MOF‐801 act as hydrogen bond donors to adsorbed H_2_O molecules.^[^ ^29^ ^]^ Another example is water adsorption by MIP‐200, a MOF based on Zr_6_ oxoclusters and a tetracarboxylate linker, 3,3′,5,5′‐tetracarboxydiphenylmethane (mdip^4‐^).^[^ ^30^ ^]^ The Zr clusters in MIP‐200 provide sites with coordinated H_2_O ligands that present hydrogen bond donor OH groups. GCMC simulations indicate that these bound H_2_O ligands act as anchors for guest H_2_O molecules which, in turn, interact with other H_2_O molecules forming clusters. These observations and conclusions were supported by ^1^H magic angle spinning NMR measurements.

**Figure 3:**
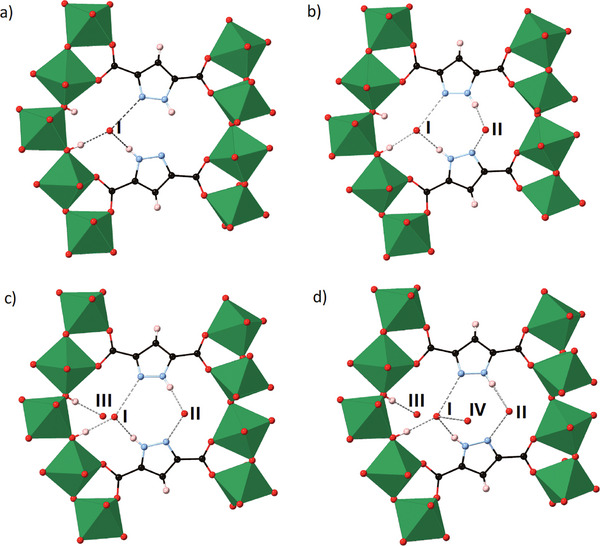
Seeding water adsorption sites in MOF‐303: a)‐d) Sequential adsorption of the first four water molecules (I to IV). Each step in the adsorption process was characterized by SCXRD studies. (O, red; H, pink; C, black; N, blue, metal polyhedra are represented in green).

An alternative approach is to use ligands that have hydrogen bonding functionality built into their structure, such as the NH groups in MOF‐303.^[^ ^31^ ^]^ A particularly elegant study by Yaghi and co‐workers combines single crystal X‐ray diffraction studies with simulations to establish how H_2_O molecules interact directly with the framework through N─H…O hydrogen bonds, via the linker 1‐*H*‐pyrazole‐3,5‐dicarboxylate, in the first instance. Subsequently, via increasing H_2_O loading, further binding sites are determined showing a series of steps in water adsorption in this MOF which appears to be a common mechanism—i) binding of H_2_O guest to framework hydrogen‐bond donors, ii) hydrogen bonding to a secondary phase of adsorbed H_2_O molecules leading to (H_2_O)*_n_* cluster formation in pores (**Figure** **3**). Thus, the role of hydrogen bonding, and hence supramolecular chemistry, in water adsorption by MOFs cannot be overestimated.

While a wealth of studies have focused on gases such as H_2_,^[^ ^13^, ^14^ ^]^ CO_2_,^[^ ^13^, ^15^, ^16^ ^]^ or CH_4_,^[^ ^13^, ^17^ ^]^ MOFs have a role to play in the adsorption and separation of other gases, including NH_3_,^[^ ^32^ ^]^ SO_2_,^[^ ^33^ ^]^ H_2_S,^[^ ^33^ ^]^ and O_2_.^[^ ^34^ ^]^ The application of MOFs for the storage of NH_3_ has proven difficult. In many cases, NH_3_ has been found to bind irreversibly to the framework or degraded it completely. Long and co‐workers reported a Cu(cyhdc) (cyhdc^2‐^ = *trans*‐1,4‐cyclohexanedicarboxylate) MOF with reversible binding of NH_3_ via insertion at the Cu‐carboxylate bond of the framework.^[^ ^32^ ^]^ The stepped adsorption isotherm of this material pointed to the phase change undergone during adsorption, as did the color change from green to blue which indicated a change in the coordination environment of Cu(II). Single crystal X‐ray diffraction (SCXRD) of the material that had been exposed to NH_3_ confirmed the phase change to a non‐porous polymer chain of Cu(NH_3_)_4_(cyhdc) wherein Cu^2+^ was coordinated to four equatorial NH_3_ atoms and two axial bridging cyhdc^2‐^ ligands bound to the metal by just one of its oxygen atoms while the other participated in stabilizing H‐bonding with the nearby NH_3_ molecules. Guest molecule coordination at the metal nodes of a host framework has been well established but the unusual phase change seen here allowed the material to reversibly adsorb NH_3_ with large uptake capacities exceeding 8.2 mmol g^−1^ and selectivity over N_2_ and H_2_. Moreover, the endothermic Cu─O cleavage offsets the exothermic heat of adsorption that often hinders the performance of NH_3_ adsorbents thereby installing intrinsic thermal management made favorable by the stabilizing H‐bonding interaction. The mechanism by which NH_3_ is adsorbed in this MOF, via a series of steps, requires stabilization by intermolecular interactions that favor the most effective adsorption of the target.

### 2.2 Complex Guests and Crystal Sponges

MOFs are not only able to adsorb simple guests, such as small gas molecules, but can also be used to host larger and highly complex molecules, including pharmaceuticals,^[^ ^35^ ^]^ dye molecules,^[^ ^36^ ^]^ and “forever chemicals.”^[^ ^37^ ^]^ All of these applications have specific requirements for successful implementation, not least of which is the ability to successfully bind the target species. As noted above, this is typically achieved through a combination of pore size/shape and intermolecular interactions between the guest and the framework. Competitive binding of targets in preference to solvent molecules is imperative in such studies.

In this section we will focus on the use of MOFs as crystal sponges,^[^ ^38^, ^39^ ^]^ an area of research that exemplifies the complexity of target that can be hosted by MOFs. The crystal sponge (CS) method was developed by Fujita and co‐workers in 2013 for SCXRD studies of guest molecules within the porous ZnI_2_/1,3,5‐triazine coordination network.^[^ ^40^ ^]^ The electron deficient π‐plane of the ligand offered a strong supramolecular interaction point for guest molecules. In this seminal study, cyclohexanone and isoprene were isolated in the host crystals and their structures were determined crystallographically. The use of a metal containing host framework allows the structure determination of compounds via SCXRD. Importantly, the CS method alleviates the need for crystallization of the guest compound making structural determination possible for liquids, oils, volatile, and scarce compounds. As a result, the CS approach is highly attractive to a wide variety of researchers and has received extensive attention. Indeed, recent reviews of the CS method provide a comprehensive discussion of the method and its applications in a range of scientific research areas.^[^ ^39^, ^41^ ^]^ As such, we aim only to discuss studies that exemplify the supramolecular interactions that are pushing forward the utility of the CS approach.

**Figure 4:**
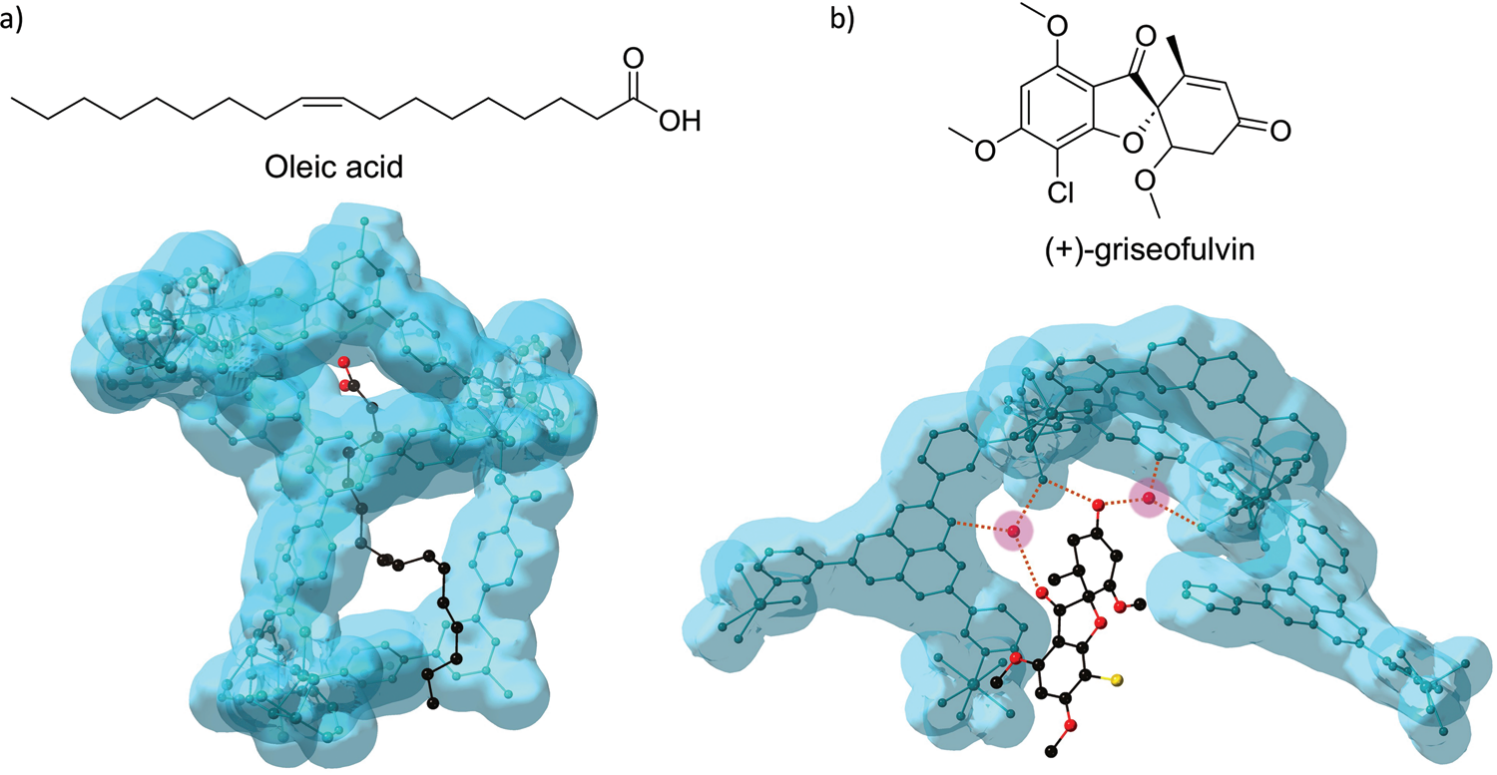
Illustrations of the guest capture modes of two crystal sponges. a) BTB‐MOF‐24 encapsulating molecules with long flexible chains, such as oleic acid, within its asymmetric sandwiching pores; and b) Co‐3TPH using its adaptable water network to position (+)‐griseofulvin with both host–guest and host–solvent–guest interactions (solvent highlighted in pink). The parent MOF frameworks are represented by a translucent, blue van der Waals surface (Guest: O, red; C, black; Cl, yellow).

By removing the need for crystallization of small molecules, the CS method has allowed for the structure determination of molecules that might otherwise not form crystalline structures. Among these include compounds with long flexible chains. For this application, BTB‐MOF‐24 was constructed with H_3_BTB (H_3_BTB = 1,3,5‐tris(4‐carboxyphenyl)benzene) and a novel rod‐like iron‐based SBU.^[^ ^42^ ^]^ Importantly, this MOF featured narrow channels and different sized cavities sandwiched between two BTB^3−^ ligands (3.4 and 6.0 Å). Because of these unique structural and chemical properties, BTB‐MOF‐24 performed especially well as a crystal sponge for guest molecules with long flexible chains while minimizing their disorder in the asymmetric sandwiching cavities via interactions with the π‐system of the ligands (**Figure** **4**a).

The delicate balance of host–guest interactions for employing the CS method has made atomic‐level resolution of guest molecules difficult to achieve. Many of the reported structures are only reported at a molecular‐resolution level and require the use of crystallographic restraints and constraints. However, atomic‐resolution was achieved for a variety of guest molecules encapsulated in the MOF Co‐3TPHAP (3‐TPHAP = tris(3‐pyridyl)hexaazaphenalene).^[^ ^43^ ^]^ While most MOFs acting as crystalline sponges rely on host–guest interactions for guest encapsulation, Co‐3TPHAP also employed host–solvent–guest interactions with its adaptable internal water network. The water network was able to adapt to 14 different organic guests with diverse sizes, shapes, and functionalities and immobilize them for improved structure quality refined without the use of crystallographic constraints and restraints, including (+)‐griseofulvin (Figure 4b). One of the guests that was analyzed, dihydroartemisinin, interconverts in solution between an alpha and beta form leading to epimerization whose equilibrium is determined by solvent polarity. A crystal structure of the alpha form had not previously been reported because of the favorable packing of the beta form. However, once encapsulated in Co‐3TPHAP the alpha form was found to be more predominant suggesting that the pore environment mimicked a polar solvent. While the polar groups of the guest compounds were important for uptake and alignment with this strategy, it was still successful for guests that had hydrophobic groups as well. The hydrophobic center of the pores accommodated these groups while the water network adapted to provide preferable host–solvent–guest interactions.

An area of focus has been the development of homochiral MOFs for the separation of enantiomers.^[^ ^41^ ^]^ An excellent demonstration of this approach was reported by Yaghi and co‐workers^[^ ^44^ ^]^ who employed the chiral framework, MOF‐520, as a host for a variety of molecules of varying complexity, from simple primary alcohols to plant hormones, such as gibberellins which contain eight stereocenters. The utility of the approach was demonstrated by the successful isolation and absolute structure determination of a specific enantiomer of jasmonic acid from a racemic mixture. The absolute configuration of this enantiomer had previously only been inferred from derivatives. The approach successfully used the framework chirality as a reference during structure determination which allowed for assignment of absolute configuration for highly complex molecules more readily than would be possible by other approaches.

A related approach used a chiral framework material, [Ni(S‐indoline‐2‐carboxylate)(4,4′‐bipy)(H_2_O)][NO_3_] termed CMOM‐5, to bind various target chiral guests incorporating alcoholic functional groups. The MOF showed enantioselectivity binding driven by MOF–guest hydrogen‐bonding interactions.^[^ ^45^ ^]^ A particularly elegant study by Zaworotko and co‐workers^[^ ^46^ ^]^ evaluated the range of interactions between a host framework and chiral guests through SCXRD analysis and Hirshfeld surface analysis,^[^ ^47^, ^48^, ^49^ ^]^ which provided valuable tool for the appreciation of the contributions of different host–guest interactions. Although it is easy to focus on simply measured interactions, for example traditional hydrogen bonds (e.g., O─H…O hydrogen bonds), host–guest behavior is rarely as simple as an individual interaction. Hirshfeld analysis allows an appreciation of the full range of interactions and, in the example reported, both π–π and C─H··· π interactions were important in positioning the guests within the framework.

Monte Carlo calculations can also be employed to understand chiral recognition processes in MOFs as in the case of a 3D MOF based on Cu(II) cations and a tripeptide linker (Gly‐L‐His‐Gly (GHG)) that was used for the enantioselective separation of methamphetamine and ephedrine.^[^ ^50^ ^]^ The simulations evaluated the role of hydrogen bonds between the framework and the guests and recognized the contributions of both strong and weak interactions, in particular, the energetics of diastereomeric adducts. Although not strictly an example of a crystal sponge, appreciating and evaluating the role of framework–guest interactions was vital to understanding the behavior of the whole system, host and guest.

## 3 Reactivity in and of MOFs

By virtue of their permanent porosity, high surface area, tunable composition, and periodic nature, MOFs have found interest as platforms to host, partake in, or study a wide variety of reactions. Confinement of reactive species within a crystalline matrix provides opportunities to influence their reactivity through numerous supramolecular effects. Various aspects of reactivity in MOFs, including catalysis, have been the subject of several recent reviews.^[^ ^51^, ^52^, ^53^ ^]^ This section focuses on the underlying supramolecular themes pertinent to influencing and understanding reactivity in MOFs. Here we initially introduce some of the supramolecular effects that govern or influence reactivity within MOFs, and ways in which reactions can be studied. The variety of reactive sites available within frameworks will then be discussed, highlighting some salient examples and the supramolecular effects which influence them.

### 3.1 Supramolecular Themes and Reactivity within MOFs

When carrying out reactions in glassware, one typically assumes that the reaction vessel has little influence over the outcome of a reaction. When carrying out reactions in MOFs, the framework can be considered to be a reaction vessel, or a “crystalline flask”. However, MOFs have well‐defined structures at the molecular level with periodic arrays of functional groups, pores, and channels with defined geometries and non‐uniform guest‐accessible space. It is unsurprising, therefore, that we cannot assume framework innocence when reactions are performed within the confinement of MOFs. Such confinement of reactive species within well‐defined chemical environments though their heterogenization within MOFs therefore provides opportunities to influence (and study—see section 3.2) their reactivity.

The geometry of pores and channels can influence diffusion of guests, reactants, and products through the MOF and should be considered when selecting the optimal framework for a particular reaction. For example, diffusion of products from the framework is desirable when reactions are performed within a MOF, in contrast to crystal sponge studies where leaching of encapsulated catalysts should be prevented. Molecular sieving effects may be observed in reactions within MOFs where pore access to substrates, reaction intermediates, or products is governed by their size compatibility with the pore dimensions. The MOF Mn_3_[(Mn_4_Cl)_3_(BTT)_8_(CH_3_OH)_10_]_2_ (H_3_BTT = 1,3,5‐benzene‐tristetrazol‐5‐yl) catalyzes the cyanosilylation of carbonyl substrates at Lewis acidic Mn(II) sites within its 10 Å wide pores.^[^ ^54^ ^]^ In the reaction with Me_3_SiCN, 90% conversion of 1‐naphthaldehyde (dimensions 9.7 × 8.4 Å^2^) to the corresponding cyanosilylate occurs within 9 h. However, conversion yields for the corresponding reactions with the larger reactants, 4‐phenoxybenzaldehyde and 4‐phenyl‐benzaldehyde (molecular dimensions of 13.3 × 7.3 and 13.1 × 6.7 Å^2^, respectively), are below 20% within the same timeframe, suggesting that the MOF pores are too small to comfortably accommodate the required transition state geometry. Limitations of pore geometry can also result in different products being preferred in heterogeneous systems compared to their homogeneous counterparts.^[^ ^55^ ^]^ Pore topology has also been exploited to allow a guest to be assembled within the pore from reactants that are small enough to pass through the pore windows, and then intentionally trapped within the framework due to the larger size of the assembled guest, this approach is termed “ship‐in‐a‐bottle” encapsulation.^[^ ^56^ ^]^

The nature of the pore environment and interactions between guest(s) and the framework have the potential to effect product selectivity. Long and coworkers report the investigation of the effect of pore hydrophobicity on product selectivity for the oxidation of cyclohexane to cyclohexanol and its overoxidation to cyclohexanone in a series of Fe‐based frameworks. Local hydrophobicity enhanced cyclohexane uptake which increased its local concentration around the catalytic Fe^2+^ centers and minimized overoxidation to improve alcohol:ketone selectivity.^[^ ^57^ ^]^ Increased selectivity has also been reported for systems where transition state stabilization and side‐reaction suppression was provided through interactions with pore functionality,^[^ ^58^ ^]^ or where MOF cavities decorated with chiral functionalities were able to influence enantiomeric excess for a reaction performed within the pores.^[^ ^59^ ^]^

The periodicity of MOFs allows for the uniform distribution and spatial isolation of identical reactive centers or encapsulated guests throughout the framework, facilitating site‐isolated reactivity. For example, when the catalyst [Rh(dppe)(COD)]^+^ is encapsulated within the pores of anionic framework ZJU‐28, it initially exhibits similar catalytic reactivity to its solution‐based counterpart, but exhibits greatly enhanced recyclability owing to suppression of catalyst decomposition pathways by matrix isolation.^[^ ^60^ ^]^

### 3.2 Studying Reactions in MOFs

Understanding reaction mechanisms, the identity of reactive species, and whether reactivity within MOFs differs from solution‐state analogues is important for guiding the synthesis of the next generation of functional reticular materials. The reactive species or encapsulated guests within frameworks can be studied using a variety of techniques, including SCXRD, PXRD, pair distribution function analysis, X‐ray photoelectron spectroscopy, and solid‐state NMR (ssNMR). Examples of these techniques can be found throughout the subsequent section.

**Figure 5:**
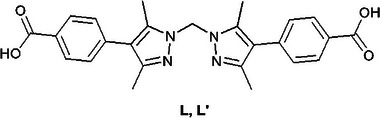
The ligand bis‐(4‐carboxyphenyl‐3,5‐dimethylpyrazolyl)methane that illustrates the strategy of incorporating binding sites for post‐synthetic incorporation of reactive metal sites to MOFs. In the case of this ligand a MOF, MnMOF‐1, is formed in which vacant *N*,*N*′‐chelation sites are retained that can be subsequently metalated.

SCXRD is the “gold‐standard” of structural determination and, where reactive species are organized in a crystalline arrangement, is a powerful technique for elucidating the nature of reactive species and their mechanisms. Study of reactivity using SCXRD requires the system to retain crystallinity throughout the reaction process and proceed with high levels of conversion since SCXRD provides an average structure. Young et al. set out guidelines for the selection of MOFs that are well‐suited to enabling detailed structural analysis of internalized reactive moieties.^[^ ^61^ ^]^ These frameworks, or reticular materials, should i) occupy a low‐symmetry space group to reduce structure disorder over special positions/symmetry elements, ii) possess structurally flexible linkers which can accommodate a change in geometry without undue mechanical stress on the framework which could result in loss of long‐range order, and iii) not have a deleteriously high density of reactive sites in order to prevent blocking of pores and consequently poor conversions. One MOF which has seen particular success in its use for the study of reactions within its framework is MnMOF‐1. MnMOF‐1 has the formula [Mn_3_L_2_L′] (L = bis‐(4‐carboxyphenyl‐3,5‐dimethylpyrazolyl)methane where L = L′, but L′ possesses a vacant *N*,*N*′‐chelation site, **Figure** **5**). The chemical robustness of the MOF combined with the conformational flexibility of the linker has allowed MnMOF‐1 to be used to bind various spatially isolated reactive metal complexes and obtain “snapshots” of their reactivity via SCXRD.^[^ ^62^ ^]^ Reactivity of MnMOF‐1 will be discussed in detail in Section 3.3.3.

### 3.3 Reaction Sites in MOFs

Reactions may take place on the surface of MOFs, within the pores, or at reactive sites on the framework itself. The precise location of a reactive site affects a number of factors, including accessibility to reagents, stability of the framework and reactive species, selectivity, and leaching. Surface reactivity of MOFs is largely beyond the scope of this review and will not be addressed here. A non‐exhaustive selection of reactions occurring at nodes, encapsulated species, framework‐bound metal sites, and linkers are discussed below, and references to supramolecular effects observed in or governing these systems are highlighted. For further information on catalysis within MOFs,^[^ ^63^ ^]^ study of reactivity in MOFs,^[^ ^62^ ^]^ or the chemistry of specific functionalities,^[^ ^52^, ^53^, ^64^, ^65^ ^]^ readers are directed to various reviews of these areas.

#### 3.3.1 Reactivity at Nodes

Far from simply being structural components, nodes play a multifaceted role in defining the properties and functions of MOFs. Robust nodes with strong metal–ligand bonds, for example the UiO series of MOFs,^[^ ^66^ ^]^ and systems with highly connected nodes^[^ ^67^ ^]^ enhance framework stability.^[^ ^68^ ^]^ On the other hand, many MOF nodes are inherently reactive due to the presence of coordinatively unsaturated metal sites originating from removal of coordinating solvent molecules (activation) or the presence of coordinative defects (missing linkers or nodes).^[^ ^69^ ^]^ Unsaturated metal centers or electron deficient groups within MOFs are able to act as Lewis acidic sites which may be exploited in adsorption or catalysis applications. MOFs with open transition metal sites have also seen application in redox catalysis.^[^ ^70^ ^]^

Lewis acidity can enhance gas adsorption through guest–framework interactions. The MOF HKUST‐1 contains Cu^2+^ paddlewheels in which each copper atom binds four oxygens from four benzene‐1,3,5‐tricarboxalate linkers and is apically capped by one water molecule. Activation by soft thermal treatment removes the coordinated water molecules to leave a coordinatively unsaturated sites capable of binding guest molecules, including CO.^[^ ^70^ ^]^

**Figure 6:**
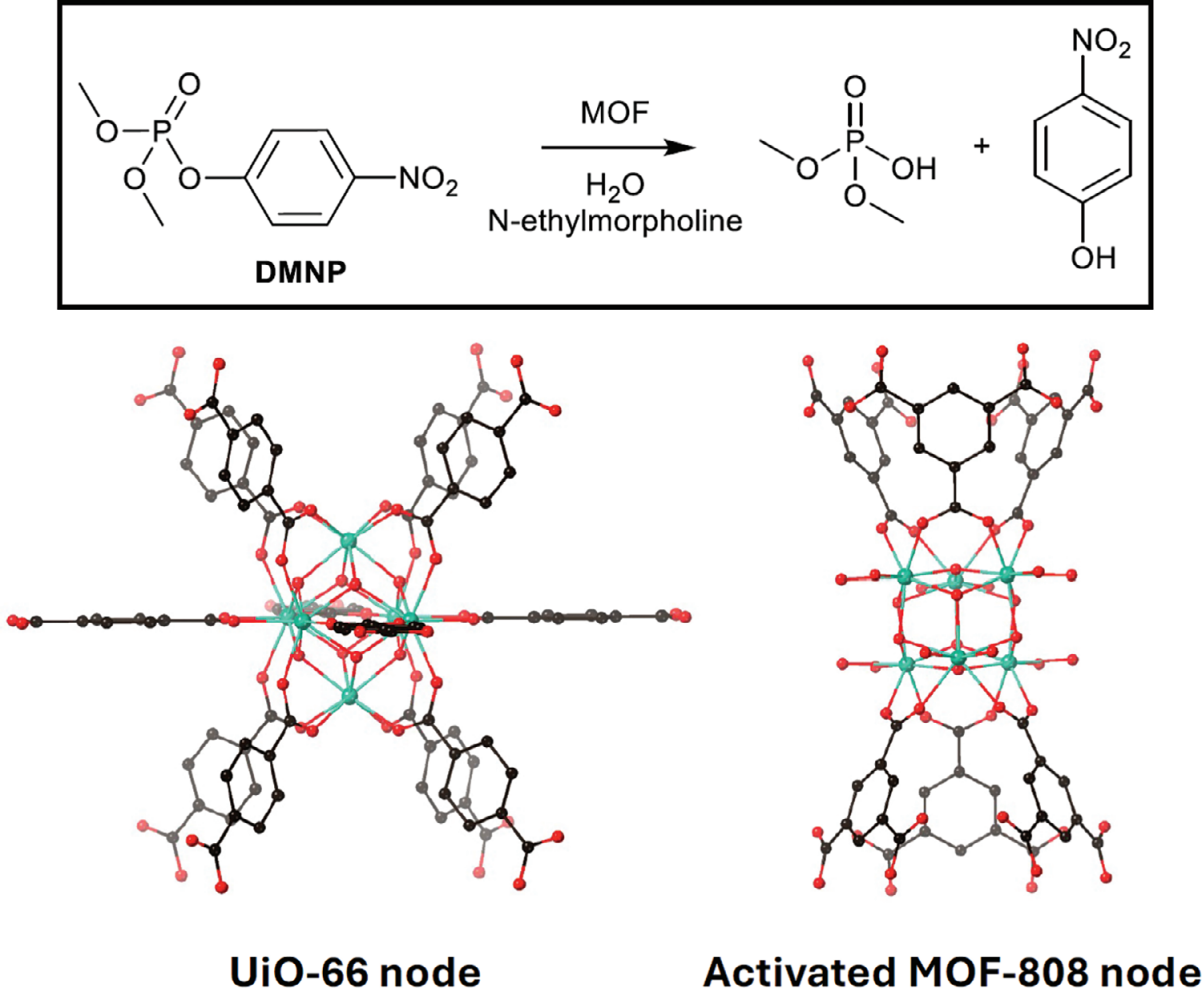
The nerve‐agent simulant dimethyl 4‐nitrophenylphosphate (DMNP) is hydrolyzed at both UiO‐66 and activated MOF‐808 nodes. Faster rates of reaction at (ii) are attributed to the increased number of node‐bound waters and reduced steric crowding. (Zr, teal; O, red; C, black).

Lewis acidic MOF nodes have been used to catalyze a range of transformations including cyclisation reactions,^[^ ^71^ ^]^ hydrolysis,^[^ ^72^ ^]^ dehydrations,^[^ ^73^ ^]^ as well as tandem reactions.^[^ ^74^ ^]^ Comparisons may be drawn between M*_x_* O*_y_* MOF nodes and metal‐oxide cluster catalysts, such as polyoxometalates, although MOFs offer a distinct advantage in that their fixed position within a framework prevents aggregation which is typically observed with metal‐oxide clusters. MOFs containing Lewis acidic nodes have shown promise in the detoxification of phosphate‐based nerve agents. Owing to strongly Lewis‐acidic Zr^IV^ sites and bridging hydroxide anions, UiO‐66 hydrolyses the nerve‐agent simulant dimethyl 4‐nitrophenyl phosphate (DMNP) at the labile P─X bond with a t_1/2_ of 35 min at room temperature.^[^ ^72^ ^]^ However, due to the small pore apertures of just 6 Å, catalytic activity is limited to the external surface of the particles/crystallites. While defect‐free UiO‐66 contains Zr_6_ nodes connected to 12 linkers, the Zr_6_ nodes in activated MOF‐808 (6‐connected) bind six linkers, six waters and six hydroxide ligands. Activated MOF‐808 (6‐connected) shows remarkably enhanced performance for the same reaction over UiO‐66, hydrolyzing DMNP within just 30 s.^[^ ^72^ ^]^ The fast rate of reaction is attributed to the high number of node‐bound water molecules, since this is inversely proportional to the number of connections to the node, as well as the reduced steric crowding around the metal site (**Figure** **6**).

Modifications to MOF nodes can be made to alter their properties and thus their reactivity profile. Treating MOF‐808 with 1M HCl replaces each bridging formate group with a hydroxide and a water molecule.^[^ ^75^ ^]^ When this material is reacted with trimethylsilyl triflate (Me_3_SiOTf) the hydroxide is then replaced by an ─OTf moiety due to the oxophilicity of Me_3_Si, resulting in formation of a MOF (ZrOTf‐BTC) with node composition Zr_6_(μ_3_‐O)_4_(μ_3_‐OH)_4_(RCO_2_)_6_[OTf]_6_. ZrOTf‐BTC is strongly Lewis acidic and outperforms the homogeneous benchmark Sc(OTf)_3_ as a Lewis acid catalyst in a broad range of organic transformations, including epoxide ring‐opening reaction, Friedel–Crafts acylation and Diels–Alder reactions.^[^ ^75^ ^]^

Since coordinative defects result in Lewis acidic sites, defect engineering is an effective way to introduce or increase the number of Lewis acidic sites inside MOFs.^[^ ^69^ ^]^ An approach to engineer defects is with a mixed linker approach where a second “defective” linker with reduced connectivity is incorporated into the framework during synthesis to yield mixed‐linker isoreticular derivatives containing nodes with missing ligators. These defective frameworks display characteristics unlike those of their defect‐free counterparts due to facile mass transport and a higher density of Lewis acidic open metal sites. HKUST‐1 consists of Cu(II) paddlewheels connected by connected by benzene‐1,3,5‐tricarboxylate linkers. A defect containing analogue of HKUST‐1 with a hierarchical porous structure can be formed by incorporating the lower symmetry benzene‐1,3‐dicarboxylate linker into the synthesis.^[^ ^74^ ^]^ This material exhibits enhanced catalytic activity for the cyanosilylation of benzaldehyde to cyanohydrins due to improved mass transport and a higher density of catalytically active Lewis acidic open metal sites. More recently, selective removal of labile nodes in the heterometallic Fe─Zn─ZIF‐8 was reported by León Alcaide et al. as a method to introduce defects in order to produce a mesoporous form of ZIF‐8.^[^ ^76^ ^]^ The Fe nodes, which are unstable to hydrolysis, were removed selectively by treating with water without affecting the Zn‐supported framework backbone.

**Figure 7:**
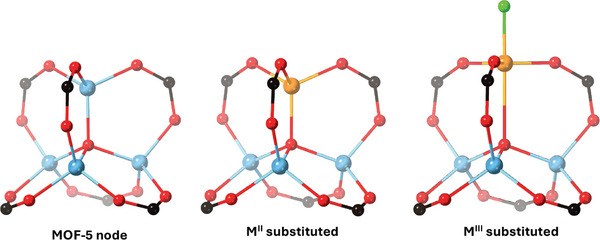
Pseudo‐tetrahedral divalent metal ions or pseudo‐trigonal bipyramidal trivalent metal ions with terminal chlorides can be substituted into Zn_4_O clusters of MOF‐5 in place of Zn to access redox‐active MOF‐5 analogues. Figure depicts a single substitution, although multiple substitutions are possible. (Zn, light blue; O, red; C, black; Cl, green; substituted metal, orange).

The redox behavior of nodes is dependent on a wide range of factors including the nature and combination of the metal ions, electronic configuration and interactions with both their inner and outer coordination sphere. Due to their unique chemical environments, MOF nodes have the potential to facilitate unusual redox chemistry. MOF‐5, also known as IRMOF‐1, has the formula Zn_4_O(BDC)_3_ (BDC = 1,4‐benzenedicarboxylate) and consists of Zn_4_O clusters linked by BDC linkers to give a cubic framework. By soaking crystals of MOF‐5 in solutions of Ti^3+^, V^2+/3+^, Cr^2+/3+^, Mn^2+^, or Fe^2+^ salts, redox active analogues of MOF‐5 are accessed with pseudo‐tetrahedral divalent metal ions or pseudo‐trigonal bipyramidal trivalent metal ions with terminal chlorides inserted into the cluster (**Figure** **7**).^[^ ^77^ ^]^ Stoichiometric redox activity is observed in these systems; outer‐sphere electron transfer achievable in the Cr^2+^ analogue Cr‐MOF‐5, and the Fe^2+^ analogue, Fe‐MOF‐5, is able to activate NO gas. MOFs with redox active nodes have seen application as redox catalysts.^[^ ^78^ ^]^ For example, Ru‐HKUST‐1 with mixed‐valence Ru^II/III^ sites exhibits exceptional olefin hydrogenation turnover.^[^ ^79^ ^]^

Nodes also play an important role in post‐synthetic modification of frameworks which is performed to obtain a desired structure or functionality. Linkers may be exchanged or incorporated by solvent‐assisted ligand exchange or solvent‐assisted ligand incorporation respectively, both of which require some level of reversibility in the metal–ligand or metal–solvent binding.

#### 3.3.2 Reactions of Encapsulated Species

Encapsulation of a guest species, for example a catalyst, allows additional functionality to be introduced to the material without synthetic modifications to the framework of the reticular material. Matrix‐isolated guests are often more stable, and their reactivities may be modified, tuned or studied.

Encapsulation of neutral species requires a specific MOF–guest combination since the guest must be small enough to occupy the pores but large enough that leaching through the pore windows does not occur. This may result in the guest blocking the pores which, in the case of encapsulated catalysts, could prevent diffusion of reagents/products through the framework. Supramolecular interactions play a part in situating encapsulated guests in a defined location and orientation. It is possible to incorporate charged guests in MOFs via ion exchange within charged frameworks, with the guest held inside the pores by virtue of complementary electrostatic interactions. Rosseinsky and co‐workers have reported the synthesis of a MOF with formula [Cp_2_Co]_3_[In_3_(BTC)_4_] (where Cp = cyclopentadienyl; BTC = 1,3,5‐benzenettricarboxylate) in a “bottle‐around–ship” type encapsulation method.^[^ ^80^ ^]^ The location of [Cp_2_Co]^+^ within the MOF pores could be crystallographically resolved, revealing that the cation sits offset from the center of the pore. Hydrogen atoms of the Cp rings hydrogen bond to O atoms of the SBUs to position the plane of one Cp ring into the guest‐accessible 3D network of framework channels. In the knowledge that cations with a similar cylindrical shape and size should be encapsulated by cation exchange in the same manner, the authors were then able to heterogenize the cationic Lewis acidic catalyst [CpFe(CO)_2_(L)]^+^ (Cp = η^5^‐C_5_H_5_, L = weakly bound solvent) through encapsulation within the pores of the same [In_3_(BTC)_4_]^3‐^ anionic framework. The integrity and reactivity of the catalyst was confirmed by its activity in Diels‐Alder reactions.

In addition to other beneficial features, encapsulation offers ways to protect against undesired reactivity. A salient example of this can be found in the heterogenization of Crabtree's catalyst, [Ir(cod)(PCy_3_)(py)][PF_6_], by encapsulation of the cationic component in the pores of the pores of sulfonated MIL‐101(Cr).^[^ ^58^ ^]^ Crabtree's catalyst, a commercially available molecular, homogeneous, catalyst for alkene hydrogenation, suffers from deactivation due to formation of inactive polymetallic hydride clusters and exhibits poor selectivity with substrates bearing ligating functionalities, such as olefinic alcohols. By contrast, the MOF‐encapsulated, heterogeneous, catalyst demonstrates enhanced activity and selectivity in the hydrogenation of olefinic alcohols when compared to its solution‐based counterpart, attributed to spatial isolation of catalytic centers in adjacent pores precluding formation of inactive clusters and suppression of competing isomerization pathway via pore‐cavity interactions. The approach described combines the benefits of the high specificity typically offered by homogeneous catalysts, with the enhanced stability and improved recyclability of heterogeneous catalysts.

This method is not limited to encapsulation of small organometallic catalysts; encapsulation of proteins within MOFs, also known as mineralization, provides a route to confer protection against denaturation while still allowing for transport of specific substrates through the pore network.^[^ ^81^ ^]^ In situ MOF growth, where the MOF grows around the protein, is a particularly appealing method for protein heterogenization since this means that protein size is not limited to the size of the pore and protects against leaching. Nevertheless, this requires the synthesis of MOFs under biocompatible conditions, rather than the high temperature syntheses in organic solvents that are more typically used in MOF preparation. Preparation of the MOF ZIF‐8 can be achieved under very mild conditions, and so it is unsurprising that many protein‐MOF composites employ ZIF‐8 matrices.^[^ ^82^ ^]^ Nevertheless, an interest in harnessing chemical interactions at the MOF‐biomolecule interface has led to novel synthetic routes to MOFs which are typically inaccessible under biocompatible conditions. Although in situ growth of HKUST‐1 is prevented in water, Cases Díaz et al. report the preparation of protein@HKUST‐1 composites by producing dense Cu‐BTC materials in water in the presence of the protein and then subsequently transforming these materials into the porous HKUST‐1 phase by immersion in EtOH.^[^ ^82^ ^]^ The presence of the MOF composite protects against proteases and offers some level of protection against organic solvents and elevated temperature.

**Figure 8:**
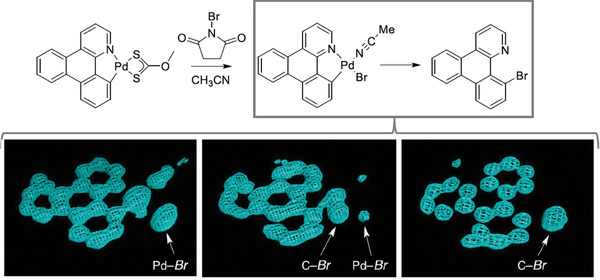
The reaction of a Pd complex encapsulated within a MOF that can be followed by studying electron density maps (shown below) in SCXRD experiments. (Adapted with permission from ref. [83]. Copyright 2014 American Chemical Society).

Encapsulation of an aryl‐Pd(II)‐L (L = ligand) complex within the pores of a MOF provided important structural evidence to aid understanding of palladium‐mediated aromatic bromination.^[^ ^83^ ^]^ When the pore isolated Pd complex is treated with *N*‐bromosuccinimide, SCXRD reveals the transient formation of an unusual Ar‐Pd(Br)(CH_3_CN) intermediate species, which then undergoes reductive elimination to release Pd and leave the brominated aryl product (**Figure** **8**). The observation of the intermediate is unusual since Ar‐Pd(X)(CH_3_CN) (X = halogen) species typically undergo rapid dimerization to insoluble Pd_2_(μ‐Br)_2_ species.

The widespread applicability of encapsulation is hindered by limitations of both the framework and encapsulated guest; guest positioning in the pores may not be precisely known, reactivity may be impeded by the encapsulated guest blocking pores, and leaching of the guest may occur. A generic approach to catalyst encapsulation seems unlikely as each catalyst will require a specific host for successful entrapment, and for a specified selectivity. However, advances in our understanding of guest capture of MOFs, as evidenced by crystal sponge studies (see Section 2.2), offer promise for a series of bespoke procedures for pairs of guests and host MOFs, fueled by an understanding of inter host–guest supramolecular interactions. In contrast, incorporation of a desired functionality into the MOF scaffold results in a reactive site with a well‐defined chemical environment, often increasing stability and ease of characterization.

#### 3.3.3 Reactivity at Supported Metal Complexes

Reactivity at metal nodes can suffer from the steric crowding of node‐bound linkers and poor stability in reactions involving significant changes in the coordination sphere (see Section 3.3.1). These issues may be circumvented by incorporating the metal supported by the ligands, whether by using a metallo‐linker or by post‐synthetically installing metal centers at vacant binding sites.

Incorporation of reactive metal sites through use of a metallo‐linker results in a uniform distribution of accessible reactive metal sites without blocking pores. The metallo‐linker, a metal complex with exodentate ligating groups, must be able to withstand the conditions of MOF synthesis. Pd(II) complexes are employed in organic synthesis as catalysts for a wide range of transformations including coupling reactions and hydrogenations. Consequently, MOFs with reactive Pd(II) sites are attractive targets, but are challenging to obtain due to facile leaching of palladium and a tendency to be reduced. Palladated linker PdCl_2_(PDC)_2_ (PDC = pyridine‐3,5 dicarboxylic acid) has been used to incorporate square‐planar Pd(II) centers directly into various heterometallic MOFs.^[^ ^84^, ^85^, ^86^ ^]^ Miguel–Casañ et al. report the synthesis of MUV‐22 which features trimeric iron‐oxo clusters bridged by six PdCl_2_(PDC)_2_ linkers to form a cubic framework with palladium complexes located in the center of the faces. MUV‐22 is an efficient heterogeneous catalyst for Suzuki–Miyaura allylation reactions, enabling C─C bond formation between alkyl, aryl and alkenyl boronates with allyl bromides.^[^ ^86^ ^]^ X‐ray photoelectron spectroscopy on the solid used in catalysis ruled out reduction of Pd(II) to Pd(0), and a hot filtration leaching test confirmed that no Pd leaches from the MOF and consequently that catalysis occurs via a MOF‐based route.

Metal centers can be incorporated into MOFs via post‐synthetic metalation (PSMet) of vacant coordination sites within the MOF which places metal centers in defined positions within the framework. Although this approach does not require the reactive metal center to withstand the conditions of MOF synthesis, there are potentially issue with incomplete metalation, uneven distribution of catalytic centers throughout the MOF which can in turn inhibit characterization by SCXRD.

**Figure 9:**
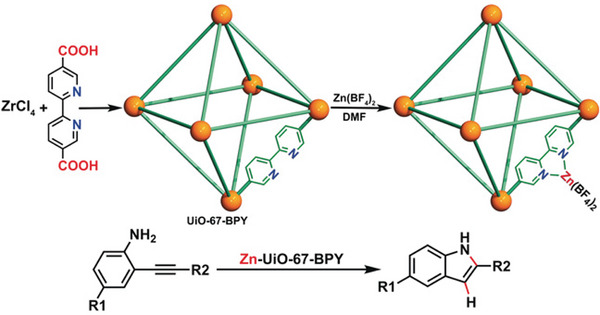
Intramolecular hydroamination of o‐alkynylanilines to indoles performed by a MOF supported Zn(II) catalyst. (Reproduced with permission of John Wiley and Sons from ref. [87]).

The UiO (Universitetet i Oslo) family of MOFs are based on [Zr_6_(OH)_4_O_4_]^12+^ nodes connected by linear ditopic carboxylate linkers: benzene dicarboxylate (UiO‐66), biphenyl‐4,4′‐dicarboxylate (UiO‐67), terphenyl‐4,4′‐dicarboxylate (UiO‐68). UiO‐type MOFs have received significant attention due to their high thermal and chemical stability, resulting in the preparation of many analogous, including that with a 2,2′‐bipyridine (2,2′‐bipy) linker, UiO‐67‐bpy. Thus, UiO‐67‐bpy contains vacant 2,2′‐bipy coordination sites which can be post‐synthetically metalated with Zn(BF_4_)_2_·*x*H_2_O to yield Zn‐UiO‐67‐bpy, in which Zn(II) coordinates to some of the uncoordinated BPY units in the UiO‐67‐bpy framework, with expected formula Zr_6_O_4_(OH)_4_(bpy)_5_[bpy‐Zn(BF_4_)_2_].^[^ ^87^ ^]^ Zn‐UiO‐67‐bpy acts as a MOF‐supported single‐site catalyst for the intramolecular hydroamination of o‐alkynylanilines to indoles, thought to occur via activation of alkynylanilines. The Zn(II) form a metastable π‐adduct which undergoes cyclisation to form the σ‐complex followed by protodemetalation to release an indole and regenerate the Zn(II) catalyst (**Figure** **9**). Notably, the products obtained via catalysis in this heterogeneous system differ from those typically obtained under similar conditions with homogeneous catalysts, attributed to the unique confined internal environment of the MOF.

A high density of reactive sites may result in blocking of the pores causing poor PSMet conversions and limiting diffusion of reagents in, and products from, the MOF. One way to circumvent a deleteriously high density of reactive sites is to use a combination of linkers where one does not have a vacant binding site. Lin and coworkers reported the synthesis of two UiO‐67 analogues containing mixtures of i) 4,4′‐biphenyl‐dicarboxylic acid with 2,2′‐bipyridine‐5,5′‐dicarboxylic acid to form mbpy‐UiO, or ii) 4,4′‐biphenyl‐dicarboxylic acid with 6‐hydroxyl‐2,2′‐bipyridine‐5,5′‐dicarboxylic acid to form mbpyOH‐UiO.^[^ ^55^ ^]^ Both MOFs undergo postsynthetic metalation with IrCl_3_ to yield mbpy‐IrCl_3_‐UiO and mbpyOH‐IrCl_3_‐UiO respectively, in which the Ir centers are bound by one bipy moiety, three chlorides, and one THF molecule. The catalytic activities of both MOFs for CO_2_ conversion to formate were investigated. mbpy‐IrCl_3_‐UiO affords a turnover frequency (TOF), based on Ir, of just 1.5 ± 0.2 h^−1^, while the hydroxyl‐functionalized mbpyOH‐IrCl_3_‐UiO exhibits a significantly higher TOF of 36 ± 2 h^−1^ under 1 atm of H_2_/CO_2_ (1:1). The improved turnover rate observed for mbpyOH‐IrCl_3_‐UiO is proposed to arise from involvement of the hydroxyl functionality in the mechanism of hydrogen transfer to CO_2_, favoring a proton–hydride transfer pathway, emphasizing the importance of local environment, and supramolecular interactions on catalytic activity.

**Figure 10:**
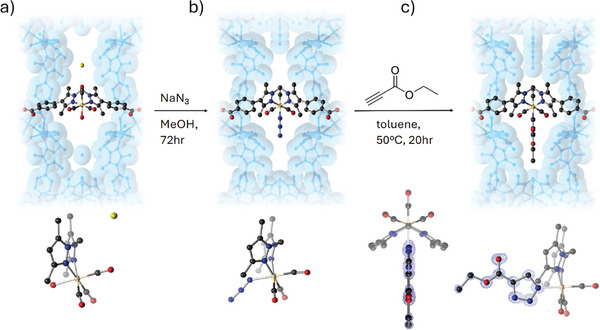
Representations of the metalated dipyrazole site in **1** at key steps in the “click” chemistry reaction scheme, with a perspective view of each complex shown below. a) **1**·[Mn(CO)_3_(H_2_O)]Br, formed via postsynthetic metalation, b) **1**·[Mn(CO)_3_N_3_], following exchange of Br^−^ for N_3_^−^, and c) **1**·[Mn(CO)_3_(ET)] (where ET = ethyl‐4‐carboxy‐1,2,3‐triazolate), the triazolate complex formed by reaction of the azide with ethyl‐propiolate, including side and top views of the *F*_obs_ electron density map associated with the triazolate. The parent MOF framework is represented by a translucent, blue van der Waals surface (C, dark gray; N, blue; O, red; Mn, beige; Br, yellow; H atoms omitted for clarity). (Reproduced with permission from ref. [89]. Copyright 2018 American Chemical Society).

MnMOF‐1 is a charge‐neutral 3D network of formula [Mn_3_(L)_2_(L′)] where L and L′ are crystallographically unique forms of deprotonated bis(4‐(4‐carboxyphenyl)‐1H‐3,5‐dimethylpyrazolyl)methane. The L form of the ligand coordinates to Mn through both the carboxylate and pyrazole donors while for L′ only the carboxylates coordinate. Thus, the framework consists of 2D layers of trinuclear Mn_3_(L)_2_ nodes pillared by the L′, resulting in channels measuring ≈8.5 × 10.5 Å which are lined with vacant, flexible, di‐pyrazole chelating units. Due to this flexibility, MnMOF‐1 can undergo quantitative post‐synthetic metalation at these vacant coordination sites with a variety of first‐ and second‐row transition‐metal ions (Mn(II), Co(II), Cu(II), Zn(II), Rh(I), Cd(II)) while maintaining crystallinity. The retention of crystallinity allows characterization of the resulting materials by SCXRD.^[^ ^62^, ^88^, ^89^, ^90^, ^91^ ^]^ Reacting single crystals of MnMOF‐1 with [Mn(CO)_5_Br] in ethanol yields MnMOF‐1·[Mn(CO)_3_(H_2_O)]Br, installing a manganese tricarbonyl moiety with a non‐coordinated Br^‐^ counter ion which sits in the pore. The authors demonstrate that subsequent reactions can be performed on the material in a single‐crystal to single‐crystal (SCSC) manner, enabling study of the coordination environment of the appended Mn center after each reaction step.^[^ ^89^, ^90^, ^91^ ^]^ Anion exchange of the non‐coordinated bromide with sodium azide forms MnMOF‐1·[Mn(CO)_3_N_3_] with a metal‐bound azide.^[^ ^89^ ^]^ Inorganic azides, such as the one in MnMOF‐1·[Mn(CO)_3_N_3_], undergo [3 + 2] cycloaddition reactions with alkyne moieties to form triazolate salts. Indeed, MnMOF‐1·[Mn(CO)_3_N_3_] undergoes reaction with ethyl‐propiolate (**Figure** **10**), and the corresponding ethyl‐4‐carboxy‐1,2,3‐triazolate complex can be identified in the electron density map.

The spatial isolation of reactive centers may be employed to perform chemoselective reactions without the requirement for protecting groups. Site‐selective transformations of dialkynes into alkyne‐functionalized triazoles are generally undertaken either by chemical protection of one alkyne or by coupling the alkyne moiety to a halogenated triazole derivative via Sonogashira coupling. The reactive Mn(CO)_3_N_3_ centers in MnMOF‐1·[Mn(CO)_3_N_3_] are periodically spaced at ≈13 Å intervals along 1D channels.^[^ ^89^ ^]^ Consequently, reactions with dialkynes smaller than the azide group spacing in the MOF (13 Å) only one alkyne is able to bind a reactive center, and so site‐selective conversion of dialkynes into alkyne‐substituted triazoles can be achieved. This selectivity is lost once the length of the dialkyne is greater than the azide…azide separation.

**Figure 11:**
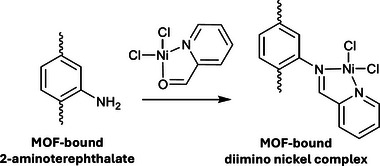
One‐pot postsynthetic grafting of a nickel‐based organometallic catalyst within the MOF (Fe)MIL‐101 via an imine condensation reaction at amines within the linkers.

Binding sites can also be installed post‐synthetically to allow for subsequent post‐synthetic metalation. (Fe)MIL‐101‐NH_2_, isostructural to (Cr)MIL‐101, is formed of trimeric iron(III) clusters linked by 2‐aminoterephthalate ligands.^[^ ^92^ ^]^ Reaction of Ni(2‐pyridine carboxaldehyde)Cl_2_ with the amines of the linker in an imine condensation reaction installs metal‐binding *N*,*N*‐chelating groups anchored to the MOF (**Figure** **11**). The MOF catalyst exhibits high efficiency in triphasic ethylene dimerization to selectively produce 1‐butene, with much higher selectively that commonly reported with molecular imino‐nickel species.^[^ ^92^ ^]^

#### 3.3.4 Reactivity at the Linker

Reactions at linkers provide routes to functionalize the MOF pores, alter mechanical properties, and even produce different topologies. Bromination of UiO‐67 analogue [Zr_6_O_4_(OH)_4_(edb)_6_]*_n_* and (edb = 4,4′‐ethynylenedibenzoate) results in the quantitative bromination of internal alkynes and consequently a mechanical contraction of the ligand, and concomitant 3.7% decrease in unit cell volume.^[^ ^93^ ^]^ Bromination proceeds stereoselectivity within the MOF to give the trans‐brominated ligand, thought to be due to geometrical constraints conferred by the framework. In a similar vein, highly reactive moieties can be stabilized within MOFs when installed post‐synthetically into linkers. Ozonolysis of the pendant alkenes on 2‐ethenylbenzene‐1,4‐dicarboxylate linkers within a UiO‐66‐type MOF installs 1,2,4‐trioxalane rings.^[^ ^94^ ^]^ Unlike in solution, the trioxalane rings remain stable under standard MOF activation conditions of heat and vacuum and can be observed crystallographically via SCXRD. Reduction with Me_2_S or oxidation with H_2_O_2_ allows for selective installation of either the corresponding aldehyde or carboxylic acid respectively.

Modifications to the pore environment can be used to optimize guest binding. The MOF MFM‐305‐CH_3_‐solv consists of [AlO_4_(OH)_2_]_∞_ chains bridged by 3,5‐dicarboxy‐1‐methylpyridinium ligands to give a 3D cationic framework with chloride counterions.^[^ ^95^ ^]^ Thermal treatment of the MOF results in demethylation of the cationic 1‐methylpyridinium moiety to give the neutral, isostructural MFM‐305 framework which exhibits enhanced adsorption and selectivity for CO_2_ and SO_2_ due to hydrogen bonds and dipole interactions between the guest gases and the framework hydroxyl, pyridyl, and aromatic C─H groups. The active polar groups in MFM‐305 mean that it could not be prepared directly; this is therefore an example of where selective bond cleavage enables the synthesis of otherwise inaccessible materials.

**Figure 12:**
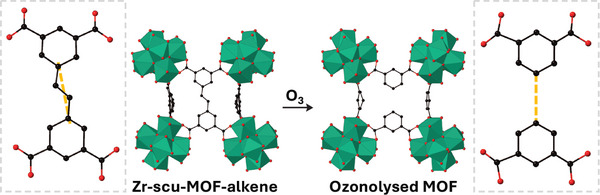
Quantitative breaking of cleavable alkene bonds in Zr‐scu‐MOF–alkene by ozonolysis results in loss of one of two circuits of connections between nodes, giving a new topology. Inserts (grey box) show the change in alignment of the phenyl rings (Zr, teal; O, red; C, black).

In recent years, the concept of “programmed disassembly” of reticular materials, including MOFs, via selective bond cleavage has been pioneered by Maspoch and co‐workers as an emerging synthetic route to otherwise inaccessible materials.^[^ ^96^ ^]^ This concept, also termed “clip‐off chemistry”, involves the selectively breaking cleavable bonds within the linkers/ligands of reticular materials to produce materials with new topologies, dimensionalities, and/or functionalities. Zr‐scu‐MOF–alkene is formed from eight‐connected (8‐c), quadrangular prismatic Zr_6_O_4_(OH)_4_ clusters and tetradentate (4‐c), rectangular 5‐[2‐(3,5‐dicarboxyphenyl)ethenyl]benzene‐1,3‐dicarboxylate linkers (**Figure** **12**), yielding a 3D framework with a 4,8‐c scu/3,3,8T132 topology.^[^ ^96^ ^]^ Two different circuits of connections exist between the clusters—the first only involving the 1,3‐dicarboxylate part of the linker, and the second involving the alkene. Exposure of Zr‐scu‐MOF–alkene to ozone results in alkene cleavage and introduction of a mixture of aldehydes and carboxylic acid groups. This step breaks one of the circuits of connection, leaving a new 3D MOF with a different underlying framework net. The reaction proceeds in a SCSC manner, allowing structural characterization of the resulting material and revealing large amounts of residual electron density in the anticipated positions of the aldehyde/carboxylic acid group. Further analysis by ^1^H NMR of the digested sample confirmed formation of trimesic acid and 5‐formylisophthalic acid linkers in the ozonolysis process.

Reactions at linkers such as those described here illustrate the importance of performing reactions inside a MOF environment. The structural configuration and chemical environment provided by individual MOFs plays a vital role in controlling chemical reactions. Indeed, the supramolecular environment, specific interactions in combination with the geometric properties of the host, play a pivotal role in developing this area of chemistry.

## 4 Mechanically Interlocked Molecules in MOFs

As explained in the previous sections, achieving a deeper understanding and precise control over the synthesis of MOFs has paved the way to the design of new materials. In terms of host–guest properties of MOFs it is clear that significant effort has been made, with great success, into developing MOFs that have specific adsorption properties. In the previous sections we have discussed the role of supramolecular chemistry and, in particular, framework–guest interactions in determining the behavior of different MOFs in delivering crystal sponges or scaffolds for studying reactions processes. These studies typically employ a static model of the MOF, and hence the framework pores, when designing and understanding the properties of the framework material. However, the development of both multivariate MOFs^[^ ^97^ ^]^ and those with correlated disorder^[^ ^98^, ^99^, ^100^ ^]^ illustrate that a single MOF crystal can contain pores with a myriad of different shapes, geometries and chemical environments. In addition to this structural complexity, there is a growing interest in developing MOFs in which the shape and environment created by the pore can be chemically tuned or even a degree of dynamic behavior can be designed into the pore.

MOFs with flexible structures have been of significant interest since it was first established that MOFs can exhibit breathing behavior, modifying the shape and size of framework pores.^[^ ^101^ ^]^ In addition to framework breathing behavior, the ligands of MOFs are able to rotate around the internode axis^[^ ^102^, ^103^ ^]^ while remaining a component of the MOF.

Alternative pathways have been developed to influence the environment of the pores, including examples reported above in Sections 2 and 3, but strategies that modify the pore by introducing chemical flexibility, such as the functionalization of pores with flexible peptide chains,^[^ ^104^ ^]^ have also been detailed. Considering all of these different behaviors it is evident that a MOF should not be considered a static framework.

**Figure 13:**
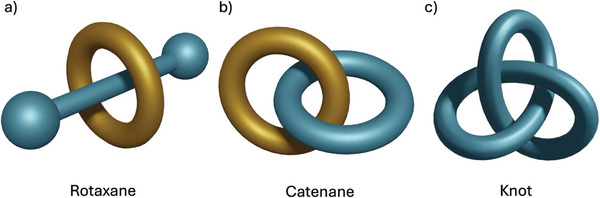
Representative examples of mechanically interlocked molecules (MIMs) that can be targeted for MOF incorporation; a) rotaxane, b) catenane, c) knot.

An opportunity to build upon the idea of MOFs with dynamic pores exploits one of the classic areas of supramolecular chemistry—MIMs. MIMs consist of multiple molecular fragments that are linked together by their topology, and their disentanglement is only possible after breaking covalent bonds.^[^ ^105^ ^]^ The link that holds the components of a MIM together is known as the mechanical bond^[^ ^106^ ^]^ and has been used to develop fascinating new molecular systems including molecular machines. The most common types of MIM include rotaxanes, in which one or more macrocycles are trapped onto a linear component (axle) by bulky substituents at its ends (stoppers) that prevent dissociation; catenanes, where two or more macrocycles are interlocked as links in a chain; and molecular knots (**Figure** **13**). The non‐covalently linked, interlocked, molecules have free movement and can respond to an external stimulus, mimicking the behavior of a molecular machine.

The dynamic behavior of MIMs at the molecular level in solution has been demonstrated but more recently research into MIMs has advanced to the solid state. This approach is interesting not only due to the inherent challenge of making such complex systems but also because the potentially controlled motion of MIMs provides a potential path to influencing the chemical and spatial composition of MOF pores. A well distributed platform is necessary to accommodate MIMs, and hence, MOFs are excellent candidates due to their high crystallinity, porosity, and stability. Specifically, the high porosity of MOFs facilitates the potential dynamic behavior of MIMs. The ability to organize and distribute MIMs in appropriate spaces that allow mechanical motion and function, such as the pores of a MOF, has been a keenly studied topic over the last decade.^[^ ^107^ ^]^ However, an understanding of how these molecules operate within the framework of a reticular material, or whether there is a synergy between them, is still a challenge.^[^ ^108^ ^]^ In this section we describe how different MIMs that have been incorporated into MOFs, explain how MIMs operate within the MOF matrix, and describe the methods used to observe their dynamic behavior. The incorporation of rotaxanes and other MIMs into MOFs inherently requires methods of incorporating macrocycles into the framework structure, either as an integral component or appended to the primary framework structure. Such strategies have been reviewed and therefore we direct readers to an excellent recent article by Fracaroli and de Rossi.^[^ ^109^ ^]^

### 4.1 Metal–Organic Rotaxane Frameworks

Initial studies that sought to incorporate MIMs into MOFs used rotaxanes leading to metal–organic rotaxane frameworks, or MORFs. Kim and co‐workers used a strategy to polymerize pseudorotaxanes (rotaxanes without stopper groups) that were functionalized such that they were capable of coordinating metals at either terminus.^[^ ^110^ ^]^ Subsequently, the same group reported the first 3D polyrotaxane network, using the higher coordination number of lanthanides to facilitate 3D network propagation.^[^ ^111^ ^]^ A related strategy was developed by Loeb and co‐workers to successfully create 2D and 3D MOFs using transition metals in combination with pseudorotaxanes composed of pyridinium^[^ ^112^ ^]^ or pyridinium N‐oxide^[^ ^113^ ^]^ axles, and crown‐ether wheels. Importantly the relative organization of the rotaxanes as MOF components could be determined by SCXRD.

The field of MORFs was developed by pioneers of MOFs, Yaghi, and MIMs, Stoddart and Sauvage, by introducing a copper template‐pseudorotaxane via the framework linkers in MOF‐1040. The arrangement of the MOF‐supported rotaxanes was determined by SCXRD confirming the presence of the MIMs. In a notable step it was demonstrated that oxidation, reduction, or demetalation of the copper from the MIM did not affect the crystallinity of the MOF.^[^ ^114^ ^]^

These structures disperse the MIMs widely and, in many examples, restrict movement of the rotaxane macrocycle. To avoid these features, Loeb and colleagues modified the MORF linker to provide rigidity, clearly separating carboxylate donors that form the extended framework. This modification allows free rotation of the macrocycle, which can be studied by ssNMR.^[^ ^115^ ^]^ Building on this approach, the same research group demonstrated that a related MOF, UWDM‐4, had sufficient free volume surrounding the rotaxane component for translational shuttling between two recognition sites.^[^ ^116^ ^]^

**Figure 14:**
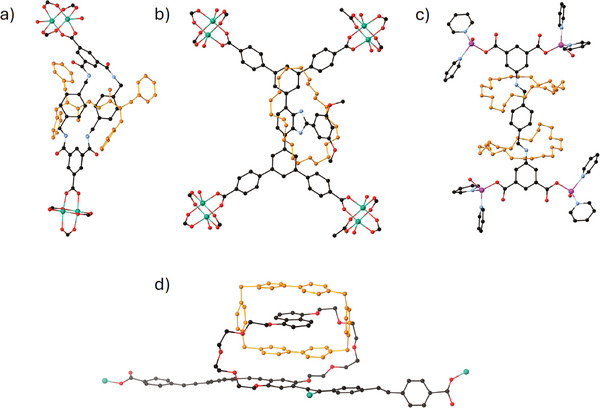
Crystal structures of MOFs containing MIMs: MORFs illustrating different MIM‐incorporation strategies: a) rotaxane perpendicular to the linkers of the MOF,^[^ ^117^ ^]^ b) MOF linker with a perpendicular appended rotaxane,^[^ ^118^ ^]^ and c) crown ether^[^ ^3^ ^]^rotaxane introducing rigidity to a flexible space,^[^ ^120^ ^]^ and d) a catenane‐containing MOF.^[^ ^123^ ^]^ One linker and its bound metal centers/clusters are shown with hydrogen atoms removed for clarity. (Cu, teal; O, red; C, black; N, light blue; Zn, purple).

An alternative approach to the development of MORFs is to position the rotaxane perpendicular to the linkers of the MOF framework (**Figure** **14**). This strategy was reported by Berna and co‐workers in the MORF known as UMUMOF (E)‐3 (Figure 14a).^[^ ^117^ ^]^ Loeb and co‐workers modified this strategy by adapting the MOF linker with a perpendicular appendage which, in turn, was used to form a rotaxane that decorated the framework by protruding into the MOF pore (Figure 14b).^[^ ^118^ ^]^ Exploiting the benefits of reticular chemistry, the study employs a functionalized version of the ligand used in NOTT‐101^[^ ^119^ ^]^ to create a T‐shaped linker capable of binding a crown ether macrocycle to form a rotaxane.

A further development realizes the potential of the mechanical bond, employed through a rotaxane, to introduce rigidity to a framework linker by encompassing a flexible linker with a crown ether wheel (Figure 14c).^[^ ^120^ ^]^ The concept of influencing the rigidity of MOFs by MIM incorporation has been implemented in a number of studies.^[^ ^121^, ^122^ ^]^

### 4.2 MOFs Incorporating Catenanes

The incorporation of catenanes into MOFs is more challenging due to their size and shape and the complexities associated with synthesizing the MIM component. This challenge is evident by the decade‐long effort required to transition from the initial rotaxane MOF structure to the first catenated MOF. Stoddart, Yaghi and colleagues reported the first catenated units in a 2D MOF, namely MOF‐1011, using donor–acceptor catenanes (Figure 14d).^[^ ^124^ ^]^ The same team reported the first 3D catenated structure within a MOF, namely MOF‐1030.^[^ ^123^ ^]^ Even though these structures were designed with long linkers, some of the acetylenic segments, which were not intended for coordination, still interacted with the SBUs, resulting in a less porous structure. In order to avoid this coordination, these acetylene groups were replaced by phenylene groups forming MOF‐1050 and MOF‐1051.^[^ ^125^ ^]^ As a result of these developments, Yaghi introduced the term “robust dynamics,” referring to the motion of an individual component that does not affect the integrity of any other linked components.^[^ ^126^ ^]^ Despite these significant advances, the formation of crystalline materials remains challenging. An alternative strategy using post‐synthetic modification, allows incorporation of catenanes into NU‐1000.^[^ ^127^ ^]^

The huge potential of incorporating MIMS, and in particular catenanes, in MOFs was demonstrated by Aida, Sato and colleagues who prepared a MOF, termed ^CTN^MOF, whose linkers are all catenanes. This MOF demonstrated remarkably low Young's moduli and a high degree of elasticity. The study demonstrates that the incorporation of MIMs in MOFs is far from a curiosity, but such materials have huge potential as squeezable porous materials.^[^ ^128^ ^]^

An alternative strategy involves catenated MOFs (termed catena‐MOFs), or self‐catenated framework structures. These materials comprise of individual nets, distinguished by the unique feature that their smallest topological rings are interlinked with other smallest rings within the same network. Yaghi et. al. reported a remarkable example of a catenated covalent organic framework.^[^ ^129^, ^130^, ^131^ ^]^ The study employs the concept of molecular catenane formation first reported by Sauvage^[^ ^132^ ^]^ in which a tetrahedral Cu(I) template is used to organize the components of the catenane via bis‐phenanthroline coordination. This “crossing point” is then used to develop the catenation throughout the extended framework structure. The implementation of a seminal strategy of catenane formation, a “classic” of supramolecular chemistry, in the formation of extended reticular materials demonstrates how reticular and supramolecular chemistry can be partnered to explore new chemistries.

### 4.3 How MIMs Operate in the MOFs Matrix

Despite significant synthetic advances in incorporating MIMs into the framework of MOFs over the past decades, a continuing challenge is our understanding of the dynamics of MIMs in the unique environment provided by the MOF. In order to study the dynamic behavior of MIMs in MOFs a variety of techniques are necessary. Using techniques that are applicable to the solid‐state environment is, of course, required and although diffraction techniques, such as SCXRD, are extremely useful for determining the structure of a compound they are often less useful for evaluating dynamics due to the averaging across individual crystals and across the timescales of the often long experiments. Advances are being made in terms of understanding dynamic processes in the solid‐state, for example through photocrystallography^[^ ^133^ ^]^ or recent advances in serial crystallography,^[^ ^134^ ^]^ but these techniques are yet to be applied to MIMs in MOFs. Thus, studies of dynamic behavior have focused on employing ssNMR which has been used to identify the rapid rotate of macrocyclic component of the MIM in MOFs^[^ ^115^ ^]^ and to demonstrate different degrees and rates of rotation depending on the size and shape of the macrocycle.^[^ ^135^ ^]^ Alternatively, photoresponsive MIMs within MOFs have been studied in which fumarate stations of a rotaxane can be converted into the corresponding intertwined maleamides in the solid state by irradiation. This chemical change in response to external stimulus provides a more permanent switch which can be readily characterized by ssNMR.^[^ ^117^ ^]^

MIMs incorporated within MOFs can display motion in different modes, such as rotational and translational motion. Rotational motion is observed for linkers in most MOFs, not just those based on MIMs, and were first studied by measuring the rotational freedom of phenylene groups in MOF‐5 using solid‐state NMR.^[^ ^102^ ^]^ Matzger and colleagues^[^ ^136^ ^]^ demonstrated the ability to control the speed of molecular motion by chemically modifying the linkers in a framework. By incorporating methylene groups, which reduced dipolar coupling, they achieved faster rotary motion. Conversely, the introduction of hydroxyl groups slowed down the dynamic rotation, illustrating how chemical modifications influence the dynamics of molecular motions. Using a related approach it has been possible to achieve dynamic control of linker motion in MIMs as a constituent component of a MOF.^[^ ^116^, ^137^, ^138^ ^]^ Thus, a subsequent study evaluated the dynamic rotation of the rotaxane wheel about the axle with three different co‐conformations.^[^ ^137^ ^]^ Translational shuttling of the macrocyclic ring of a MIM strut was similarly studied using ssNMR.^[^ ^116^ ^]^

One of the most commonly used techniques to stimulate MIMs is by switching the electronic state through the application of an electrochemical potential. This approach has been shown to be successful for the post‐synthetic transformation of MOFs, creating solid‐state molecular switches and machines.^[^ ^139^ ^]^ A similar stimulus was employed to achieve reversible redox‐switching of bistable catenanes, showing robust dynamics inside the nanopores of NU‐1000‐Fc^n+^.^[^ ^127^ ^]^ These studies illustrate the possibility to achieve controlled and predictable switching between two states within the MOF environment.

A statistical adsorption model has been used to calculate the effect of gas sorption on the MIM shuttling process in the MOF UWDM‐4, inducing a change in the configurational entropy of the shuttling wheel.^[^ ^140^ ^]^ The same MOF has been investigated using structural dynamic simulations in order to investigate the effect of structural constraints on shuttling and any potential cooperative effects.^[^ ^141^ ^]^ It was concluded that the shuttling mechanism of the MIM depends on the environment and relative position of the MIMs. Whereas well separated MIMs do not influence one another, when in close proximity competition arose between the intermolecular interactions of the MIMs and their interactions with the framework. These results highlight the importance of examining the dynamic network of intermolecular interactions when synthesizing molecular materials.

The ability to incorporate MIMs into the framework structure of MOFs has been demonstrated for a small number of MIM systems. The synthesis of such MOFs is challenging, due to the chemical and topological complexity of the MIM linkers, but these fascinating structures depict a marriage of two of the most enduring fields of modern chemistry. The structures are equally challenging to study in terms of their dynamic behavior. It is apparent that we are still in the early days of developing MIMs in MOFs, and that not only are there many more systems waiting to be discovered but also our understanding of the dynamic properties of these materials is certain to expand as more focus is brought to these exciting systems.

## 5 An Outlook

The importance of supramolecular chemistry in understanding the behavior of MOFs is self‐evident and has far reaching implications for the application of MOFs in a number of fields. Despite disparate applications and chemistry, plenty of opportunities for cross‐fertilization are present. Whether studying crystal sponges, reaction processes in MOFs or the behavior of MIMs in the confined environments of frameworks, performance is determined by intermolecular interactions between the MOF and guest molecules. A number of key challenges are prevalent in the fields discussed, many of which are pervasive in the wider field of MOFs:

- detailed structural characterization and analysis.
- understanding and quantifying framework…guest interactions.
- retention and maintenance of crystallinity in the presence of dynamic processes.
- measurement and computational analysis of dynamics within the MOF environment—for example, MIM dynamics or guest diffusion to active recognition sites.

This article describes how supramolecular interactions are integral to the behavior of MOFs and in particular their host–guest properties. The understanding of guest encapsulation within MOFs demands detailed characterization, whether studying simple guests (such as gases) or more complex molecules such as those investigated using the crystal sponge approach. This may require the energetics of an adsorption process to be quantified, which would require analysis beyond characterization by diffraction techniques. For example, the adsorption of gas molecules is often energetically quantified^[^ ^20^, ^21^, ^22^ ^]^ but such measurements are not typically employed for more complex molecules studied by crystal sponge methodology. There is an opportunity for improved understanding by extending energetic evaluation of more complex systems. More in depth computational analysis of framework…guest interactions can supplement experimental characterization, with one approach being Hirshfeld surface analysis.^[^ ^47^, ^48^, ^49^ ^]^

It is also apparent that in each of the applications described in Sections 2, 3, 4 the ability of the MOF to undergo changes while retaining integrity is an important feature. This may be simply withstanding the introduction of a new guest molecule, with or without dynamic adaptation of the framework structure. In some of the examples described above, such as studying reactive processes within MOFs, the framework can be put under considerable strain, as the reaction product requires structural adaptation to accommodate the new species. Similarly studying the motion of MIMs in MOFs can require a significant rearrangement of the framework, potentially exerting strain on the overall MOF. For this dynamic approach to be successful the MOF must be capable of adaptation.

Understanding the dynamics and structural modification of a MOF structure presents significant challenges which requires the use of multiple techniques, not least of which are spectroscopic measurements of the framework before, after and during any dynamic process. Whereas the use of a MOF as a crystal sponge requires the characterization of a MOF before and after guest capture, studying a reaction within a MOF may necessitate investigations during the reaction process. Studying MIMs in MOFs potentially also requires studies of the dynamic processes associated with the MIM and thus will require investigations beyond diffraction measurements. Due to the timescales of the measurements involved in dynamic processes various spectroscopic investigations may be required, such as time‐resolved spectroscopy^[^ ^134^, ^142^, ^143^ ^]^ or ssNMR measurements.^[^ ^112^, ^115^, ^135^ ^]^

The last feature which requires enhanced understanding is a greater appreciation of guest diffusion through the MOF. This is important for crystal sponge studies where the ability of the target molecule to diffuse to each pore throughout the crystal is paramount to achieving high loading of ordered guests for SCXRD analysis. Similarly for reactions in MOFs efficient diffusion of reagents to and from reaction sites is required for effective conversion and subsequent studies of the MOF. Our understanding of diffusion of molecules through MOFs is an ever present challenge but modelling studies are beginning to present a more detailed understanding of the processes involved.^[^ ^144^, ^145^ ^]^ Such modelling investigations present a path to greater understanding of diffusion in MOFs and are vital to developing a greater understanding of crystal sponge studies and investigations of reactions in MOFs.

Therefore, opportunities exist for improved analysis and understanding of MOFs and the role of supramolecular chemistry in their behavior. In our opinion, specific opportunities are apparent. First, more focus should be given to the thorough analysis of framework…guest interactions. It is common, and typically more straightforward, to assess readily measured interactions, such as strong hydrogen bonds. Although these may be the dominant forces in determining behavior, other interactions can be important and as demonstrated, in particular, by crystal sponge studies. The use of strategies such as Hirshfeld surface analysis provide a valuable tool for appreciation of disparate interactions. Dynamic behavior is a second feature which is not commonly studied, perhaps due to the requirement for further experiments and analysis. The use of spectroscopic techniques, including ssNMR, provide mechanisms to probe the dynamic behavior of MOFs and their guests. However, the emergence of time‐resolved diffraction studies^[^ ^95^, ^96^, ^134^, ^146^, ^147^ ^]^ has the potential to deliver insight into dynamic behavior in combination with other studies. With increased focus on these aspects of MOF chemistry and the role of supramolecular chemistry therein it is apparent that a more detailed understanding of controlled adsorption, reactivity, and dynamic behavior in the environment of MOFs will be achieved. Thus, the research highlighted in this review illustrates both the importance of supramolecular chemistry in the field of MOFs and breadth of possibilities for supramolecular concepts to be applied to the field of MOFs and reticular chemistry.

## Conflict of Interest

The authors declare no conflict of interest.
